# And Yet They Act Together: Interpersonal Perception Modulates Visuo-Motor Interference and Mutual Adjustments during a Joint-Grasping Task

**DOI:** 10.1371/journal.pone.0050223

**Published:** 2012-11-28

**Authors:** Lucia Maria Sacheli, Matteo Candidi, Enea Francesco Pavone, Emmanuele Tidoni, Salvatore Maria Aglioti

**Affiliations:** 1 Department of Psychology, Sapienza University of Rome, Rome, Italy; 2 IRCCS, Fondazione Santa Lucia, Rome, Italy; Brain and Spine Institute (ICM), France

## Abstract

Prediction of “when” a partner will act and “what” he is going to do is crucial in joint-action contexts. However, studies on face-to-face interactions in which two people have to mutually adjust their movements in time and space are lacking. Moreover, while studies on passive observation have shown that somato-motor simulative processes are disrupted when the observed actor is perceived as an out-group or unfair individual, the impact of interpersonal perception on joint-actions has never been directly addressed. Here we explored this issue by comparing the ability of pairs of participants who did or did not undergo an *interpersonal perception manipulation* procedure to synchronise their reach-to-grasp movements during: i) a *guided interaction*, requiring pure temporal reciprocal coordination, and ii) a *free interaction*, requiring both time and space adjustments. Behavioural results demonstrate that while in neutral situations free and guided interactions are equally challenging for participants, a negative interpersonal relationship improves performance in guided interactions at the expense of the free interactive ones. This was paralleled at the kinematic level by the absence of movement corrections and by low movement variability in these participants, indicating that partners cooperating within a negative interpersonal bond executed the cooperative task on their own, without reciprocally adapting to the partner's motor behaviour. Crucially, participants' performance in the free interaction improved in the manipulated group during the second experimental session while partners became interdependent as suggested by higher movement variability and by the appearance of interference between the self-executed actions and those observed in the partner. Our study expands current knowledge about on-line motor interactions by showing that visuo-motor interference effects, mutual motor adjustments and motor-learning mechanisms are influenced by social perception.

## Introduction

Contradicting the adagio “if you want something done right, do it yourself”, we continuously perform everyday life tasks with other people as we live dipped into an interactive social environment where we act in concert with others and where we are influenced by the impression others give us at first-sight. These joint-actions imply fine-tuned and smooth coordination that humans highly refine with expertise, as in the case of tangoing couples or duet playing pianists. However, interacting with others may be difficult because of the complexity of aligning oneself with the other on a common ground. Indeed, dual coordination is only achieved if co-agents act in conjunction instead of following their own strategy [1], and “mutually adjust” at some level of the planning process (intention, action plans and movement, [2]; see also [3]–[4]). Moreover, each individual has no direct access to the programming of the other's action and can only execute his own movements relying on predictive simulations of when the partner will act and what he is going to do [5].

Several processes may play a role when two people interact, in an emergent-planned continuum [6]. Ecological psychologists have applied a dynamic system approach to demonstrate that people end up spontaneously synchronizing even when they are not explicitly planning to act in concert [7]–[12] due to “entrainment processes” [13]–[14] or to the fact agents are sharing the same environment and thus follow the same environmental motor cues (affordances) and/or are influenced by similar action-perception coupling mechanisms [15].

A crucial issue in interactive contexts is that co-agents often need to perform incongruent actions with respect to the partner's ones in order to achieve the common goal. In this regard, Van Schie and colleagues [16] reported a reversal of automatic imitation effects when participants are engaged in a cooperative joint-grasping task with a virtual co-actor. Accordingly, while interference of action observation on action execution occurs when observed incongruent actions are irrelevant to the task [17]–[19] (see also [20] for a review) likely because these circumstances require inhibition of automatic covert imitation, on the contrary, complementary actions (albeit incongruent with the co-actor's ones) do not imply an additional computational cost when participants are instructed to complement the partner's movement [16]. Authors suggest [16], [21] that this flexibility in action-perception coupling may be due to associative sequence learning [22] developed during social interactions (see also [23]–[24]). However, these studies focussed on imitative and complementary actions in *joint-like* contexts where participants observe and subsequently or on-line execute their action rather than coordinate themselves with an on-line responsive partner. Furthermore, in almost all the previous studies the participant's freedom to move was very restricted or nearly absent [25]–[26]. As a consequence, studies in which two people have to mutually adjust in time and space choosing between different individual sub-goals is lacking, as well as investigations concerning the way a person adapts his behaviour to another co-agent who is himself trying to adapt at the same time (“close loop processes”, [27]).

Nonetheless, computational models have already suggested ([28], see also [3]–[4]) that the ability to properly adapt to others' behaviour during interactions might rely on the same feed-forward mechanisms supporting self-executed movement correction and motor learning. Since during interactions the behavioural output of one individual becomes also an input to the other individual, a social interactive loop is established (see also [29]). These claims parallel the finding that most of the “mirror neurons” (i.e. monkey's premotor and parietal neurons discharging both during movement execution and during the observation of similar movements performed by others [30], which are thought to be present also in humans [31]–[32]) code the outcomes of actions rather than the means by which actions are accomplished (for a review see [33]). Moreover, they suggest that others' actions may be coded in anticipatory terms [34]–[37], since their consequences would be predicted in Bayesian terms by means of simulation [38]. This would let co-agents reciprocally create “forward models” of others' behaviour just as they would do with their own motor plans [28], and would let movements corrections arise in order to adapt to others when required. However, very little is known about this issue. Similarly, the bidirectional effect of these processes on interpersonal perception has never been considered. Crucially, indeed, not only are we constantly asked to interact with others, but we do so in social contexts in which our behavior is influenced by first sight impressions, social categorizations and stereotypes; as a matter of fact, it has been shown that somatomotor- and affective- simulative neural responses are modulated by the perception of others' status, group membership and similarity [39]–[42]. For example, passive observation of motor or somatic states of a model coded far along the in-group/out-group or fair/unfair continuum reduces neural responses in affective and somatomotor cortical and subcortical nodes of the sensorimotor network of an observer [43]–[46]. Thus, observed states of others may be mapped onto our own sensorimotor system according to the degree of closeness we feel with the observed person. However, although social biases and interpersonal coding are automatic and unavoidable when interacting with others [47]–[48], their impact on covert simulation has never been investigated during face-to-face motor interactions. This seems surprising because interpersonal variables are fundamentally important in joint-action contexts and since – from the opposite perspective - it has already been shown that being involved in synchronous interactions promotes perceived similarity with others and improves altruistic behaviors [49]–[50]. Furthermore, studies on joint-attention have shown that social and emotional factors modulate the emergence of shared representations, preventing “joint” interference effects (e.g. the joint Simon effect) when the partner is perceived as non-cooperative and unfriendly or when the task requires limited interdependence between participants [51]–[52].

In the present study we aimed to investigate whether the ability to coordinate with a partner and the kinematics of a joint reach-to-grasp action are modulated by co-agents' reciprocal interpersonal perception. We studied the ability of two individuals who did not know each other in advance to learn how to coordinate themselves in grasping two objects either via “imitative” or “complementary” movements in order to maximize economic pay-off. Two different interactive conditions were investigated, namely i) a *Guided interaction*, requiring reciprocal partners' adjustment in time only: each individual was informed on where to grasp the object and instructed to be synchronous with his partner, and ii) a *Free interaction*, requiring both time and space adjustments: participants were asked to on-line re-model their individual sub-goals to achieve a joint-goal without knowing what their partner was going to do. Further, in two different groups of participants, interpersonal perception was either left neutral or negatively biased.

We specifically hypothesized that inducing a negative interpersonal perception would differently affect the co-agents' coordination ability in Free and Guided interactions and that this interpersonal manipulation might also be reflected in movement kinematics. Moreover, the analysis of differences in the kinematics of imitative and complementary actions allowed us to investigate the presence of “interference effects” [19] between co-agents' movements, which we expected to be absent in neutral conditions on the base of previous literature on joint-actions [16], [21]. Importantly, the behavioural and kinematics analyses of the joint-grasping task were performed after having assessed the reliability of the interpersonal perception manipulation.

## Materials and Methods

### Participants

Twenty-eight male participants took part in the experiment and were randomly assigned to two groups (each made of seven pairs), i.e. “Neutral group” (NG), age 24.2±2.9; “Manipulated group” (MG), age 23.7±4.5. Based on previous findings indicating that the impact of an unfair partner's behaviour is stronger in men compared to women [46], only male participants were selected. All participants except one per group were right-handed as confirmed by the Standard Handedness Inventory [53]. All participants reported normal or corrected-to-normal vision and were naive as to the purpose of the experiment. Participants gave their written informed consent to take part in the study, received a reimbursement for their participation and received a debriefing on the purpose of the experiment at the end of the experimental procedure.

### Ethics Statement

The experimental protocol was approved by the ethics committee of the Fondazione Santa Lucia and was carried out in accordance with the ethical standards of the 1964 Declaration of Helsinki (Prot. CE-PROG.282-40: In date 09.07.2010 the Ethical Committee of the Fondazione Santa Lucia examined the proposal of the study “Kinematics and neural correlates of social and emotional interactions in realistic contexts”; the Committee approved the above-mentioned study).

### Stimuli

Each participant had to reach and grasp one bottle-shaped object (30 cm total height) constituted by two superimposed cylinders with different diameters (small, 2.5; large, 7.0 cm) placed next to the centre of the working surface, 45 cm away from the participants and 5 cm on the right of the midline. In order to record participants' touch-time on the bottle, two pairs of touch-sensitive copper plates (one for each cylinder) were placed at 15 cm and 23 cm of the total height of the object.

Auditory instructions concerning the movement to be executed were delivered synchronously to both participants via headphones. The instructions consisted in three sounds having the same intensity (4 db) and duration (200 ms) but different frequency: i) “high-pitch”, 1479 Hz, ii) “low-pitch”, 115.5 Hz, iii) “whistle”, 787.5 Hz.

### Apparatus

**Figure 1:**
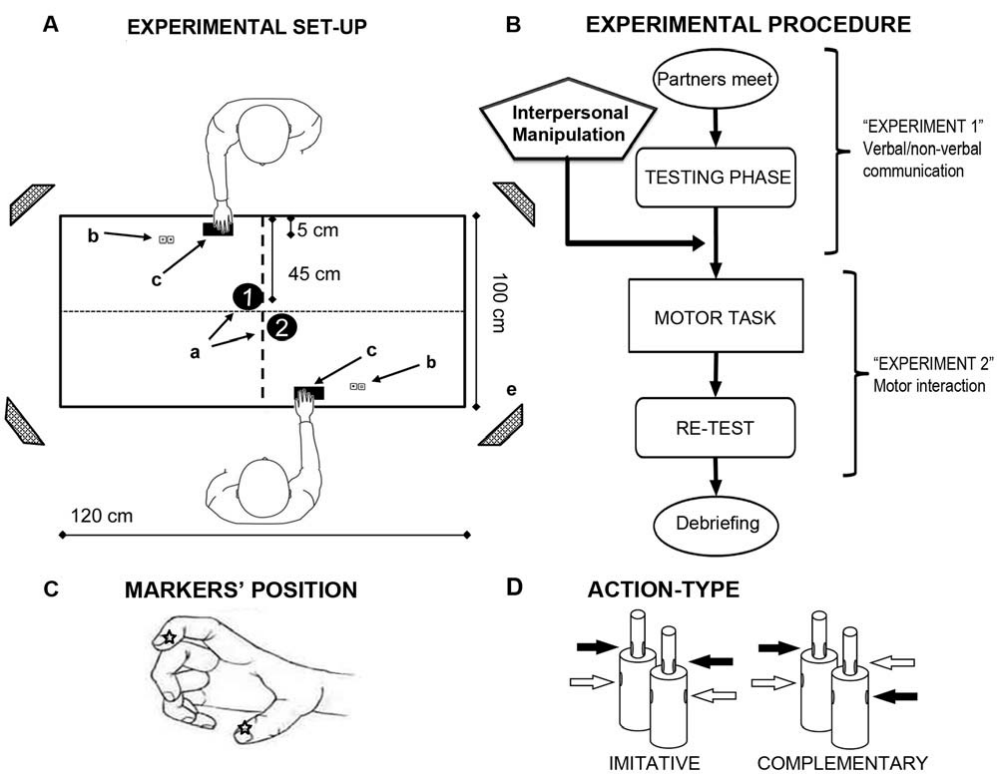
Set-up and experimental procedure. Panel A: Top-view of the experimental set-up. Participants sat one in front of each other, with their right hand placed on the Start-button (c), and reached-to-grasp their bottle-shaped object (a) trying to be as synchronous as possible. A pair of green/red LED (b) was placed in front of each participant to give GO-signals and feed-back signals about pair's performance. Panel B: flow-chart of the experimental phases. Panel C: position of the infrared reflective markers on the participants' right hand; kinematics has been recorded from the thumb (ulnar side of the nail) and index finger (radial side of the nail). Panel D: schematic representation of the Action-type participants were required to perform during the Free Interaction condition. Importantly, in imitative trials they had to perform the same movement (both grasping either “up” or “down”) while they had to do the opposite during complementary trials.

Two participants were seated opposite to each other in front of the working surface, a rectangular table of 120×100 cm. Before each trial, each participant rested his right hand on a starting button placed at a distance of 40 cm from the bottle-shaped objects and 10 cm on the right of the midline, with the index finger and the thumb gently opposed. For each subject, the GO signals as well as the feedback signals were provided via a green/red LED placed next to the partner's hand starting position (Figure 1).

Infrared reflective markers (5 mm diameter) were attached to participants' right upper limb on the following points: i) thumb, ulnar side of the nail; and ii) index finger, radial side of the nail. Movement kinematics was recorded (sampling rate 100 Hz) using an ELITE motion analysis system (Bioengineering Technology & Systems [B|T|S]). Four infrared cameras with wide-angle lens placed about 100 cm away from each of the four corners of the table captured the movement of the markers in 3D space. The standard deviation of the reconstruction error was always lower than 0.5 mm for the three axes. Kinematics was computed for both participants at the same time.

### Procedure

In order to make the social manipulation reliable, participants were told they would take part in two separate experiments on: i) “verbal and non-verbal communication” (“Experiment 1”, i.e. Interpersonal Manipulation); and ii) “motor interaction” (“Experiment 2”, i.e. Joint grasping Task).

Participants were told (as cover story) that the first experiment aimed at studying the correlation between personality traits and communication-styles used by people to describe themselves to strangers, while the second experiment aimed at studying motor coordination learning. Importantly, participants were led to believe the two experiments were not directly linked to each other.

#### Interpersonal Manipulation

Participants were asked to complete a series of personality tests: a 125-item version of the Temperament and Character Inventory (TCI, [54]); the Reading the Mind in the Eyes Test, [55]; the Personal Norm Reciprocity, (PNR, [56]); a test on Leadership (scale created from the International Personality Item Pool, IPIP [57]); and a pen-and-pencil questionnaire in which they were asked to describe their personal background (e.g., family, childhood, education), future perspectives (e.g., their plans within three years), hobbies and personality (e.g., “list three of your gifts and flaws”). Once they had finished compiling these tests, participants were given the partner's questionnaire and were asked to read through it and judge through Visual Analogue Scales (VAS1, Judgments on partner personality – Pre-interaction): (i) several traits of their partner's personality (i.e., “Based on your impressions, how much do you rate your partner a self-confident/ easy/ friendly/ original/ mature/ intelligent/ calm/ agreeable/ sincere person?”), (ii) the perceived similarity with the partner, and (iii) the level of cooperation quality they expected to reach if asked to interact with him. In addition, participants completed a 25-items self-referred version of the BIG-5 personality questionnaire [58]–[59] and a modified version of the same questionnaire referred to their perception of the partner (BIG-5 Other-Pre).

After having completed the personality testing, half of the sample (the Manipulated group, MG) received a negative “false-feedback” about the partner's judgements (See Figure S1). More specifically, MG participants were led to believe their partner did not esteem their interests and personality (“self-esteem threatening manipulation” procedure, [60]). Immediately after this manipulation, participants were asked to assess along VASs the subjective impact of the “false-feedback” (VAS2 - Reaction to manipulation): VAS2 included a key-question concerning a re-rating of the level of cooperation quality they expected to reach if asked to interact with their partner. No feedback was given to the Neutral group.

#### Joint grasping Task

During the whole experiment, participants' task was to grasp as *synchronously as possible* the bottle-shaped object in front of them, executing different individual movements according to auditory instructions. The instructions could either be: i) a whistle, implying they would have to perform a *Free interaction*; or ii) a high- or low-pitch sound, implying they would have to perform a *Guided Interaction*. In Guided interactions the sound would specify which part of the object they had to grasp: a low-pitched sound would mean “grasp the lower part” of the bottle-shaped object, while a high-pitched sound would mean “grasp the upper part”. Given the bottle-shaped object dimensions, grasping the lower part would imply a whole-hand grasping (“Gross grasping”), while grasping the upper part would imply a finer movement performed with the thumb-index finger only (“Precise grasping”). Conversely, during the Free interaction condition, both partners were free to grasp either the upper or the lower part at will. However, in different blocks (i.e., “Complementary” or “Imitative”), each participant had to do the opposite/same movement with respect to his partner; the opposite/same instruction to be followed in the free interaction condition was given at the beginning of each block. We monitored the movements to ensure that partners did not implicitly agree on a consistent strategy (e.g., one always grasping the top and the other the bottom).

On each trial, the LED visible to each participant was turned off to alert about the impending whistle/sound instruction go-signal. Upon receiving the synchronous auditory instruction participants could release the Start-button and reach-to-grasp the object. Given the simultaneous delivery of the auditory instruction, no explicit leader/follower role was induced. Thus, each participant had to monitor the partner's movement and adapt to it accordingly. Participants knew they would always receive the same kind of instruction of their partner (sound/whistle to both) and that in the Guided interaction condition same or different sounds could randomly be delivered to them. At the end of each trial, participants received a feedback (the green/red LED turned on) about their performance as a couple (win/loss trial). A win trial needed that both participants followed their own instructions and achieved synchronicity in grasping the objects. The action was considered synchronous when the time-delay between the partners' index-thumb contact-times on their bottle fell within a given time-window which was narrowed or enlarged on a trial by trial basis according to a stair-case procedure. Thus, the window for considering synchronous a grasp became shorter as participants got better in the task and longer if they failed in three consecutive trials; as a result, this procedure allowed tailoring the time-window to assess grasping synchronicity on the peculiar ability shown by each couple. Participants knew their monetary reward would depend on the number of wins accumulated during the experimental sessions. Previous to any recording of the motor task, participants practiced the task as long as they needed to achieve an errorless association of whistle/high-pitched/low-pitched sounds with the correct instruction; moreover, a preliminary block constituted by 10 whistles and 12 sounds (requiring either imitative or complementary response, counterbalanced between pairs) was provided in order to let participants better familiarize with the task. Then, participants performed two sessions, each comprising one Complementary and one Imitative block delivered in counterbalanced order in the different couples. Each block consisted of 66 trials divided in 3 sub-blocks of 10 Free interaction (whistle) plus 12 Guided interaction (sounds) trials. The order of Free and Guided instructions was counterbalanced in the different couples. In the Free interaction conditions, the instruction to perform imitative or complementary actions was given at the beginning of the block. Unbeknownst to the participants, this instruction implied consistent imitative or complementary actions also in the guided interaction condition in 10 out of 12 sounds for each sub-block. In the 2 additional Guided trials for each sub-block, the sounds instructed each member of the couple to perform a type of action (complementary or imitative) non consistent with the rest of the block: these two “odd trials” aimed at making the partner's movements less predictable and were excluded from the analyses. Stimulus presentation and randomization were controlled by E-Prime1 software (Psychology Software Tools Inc., Pittsburgh, PA).

#### Manipulation-check and debriefing

At the very end of the experiment, all couples completed again the VAS ratings regarding judgements on partner's personality (VAS3 - Judgments on partner personality – Post-interaction) and the BIG-5 personality questionnaire referred to the partner (BIG-5 Other-Post). Finally, participants in the MG were explicitly asked whether they believed or not that the false-feedback was actually given by their partner (manipulation-check procedure). At the end of all experimental procedures, all participants were debriefed.

### Data handling

Only correct trials were entered in the behavioural and kinematics analyses.

We considered *as behavioural measures*:

1. Reaction Times (RTs), i.e., time from the instant at which participants received the auditory instruction to Start-button hand release, as measures of movement preparation timings;
2. Grasping Synchronicity, i.e., absolute value of time delay between the partners' index-thumb contact-times on their bottle, i.e., [abs (sbjA's contact-time on the bottle – sbjB's contact-time on the bottle)]; please notice that “contact-time” is defined as the time from the GO-signal (which is common for both participants) to the instant of participants' index-thumb contact on their bottle;
3. Accuracy, i.e., number of movements executed according to participants' instructions;
4. Wins, i.e., number of correct trials where Grasping synchronicity was below the time-threshold (corresponding to the amount of money earned at the end of the experiment).

For each of the above-mentioned behavioural measures we calculated the individual mean in each condition. These values were entered in a mixed ANOVA (see below). With regard to RTs, we calculated individual mean and individual variance of the RTs recorded for each condition (see Table S2), the latter being considered an index of movement preparation variability. Moreover, we calculated the trial-by-trial time-delay between partners' Reaction Times (Start Synchronicity, “Diff_RTs”); the analysis on this index was aimed at testing whether participants would end up automatically synchronizing (“entrain”) their RTs (i.e., their movement preparation timings) although not explicitly asked to do so.

The ELIGRASP software package (B|T|S|) was used to analyse the data and provide a 3-D reconstruction of the marker positions as a function of time. The times of Start-button hand release and the index-thumb contact-times on the bottles were used to subdivide the kinematic recording with the aim of analysing only the reach-to-grasp phase, i.e., from the instant the quickest participant released the Start-button to the instant the slowest participant touched the bottle.

As *kinematic measures* we focused on the pre-shaping components of the reach-to-grasp [61]–[62] and analysed:

1. the index-thumb maximum 3-D Euclidean distance (maximum grip aperture, “MaxAp”);
2. its variance (Var_MaxAp), as an index of variability in following the typical pre-shaping pathway of each individual.

We selected maximum grip aperture kinematics because it has been shown to be an index sensitive to the ultimate goal of the grasping and to the social context [63]–[68].

Each behavioural and kinematic value that fell 2.5 SDs above or below each individual mean for each experimental condition was excluded as outlier value (on average, 1.4% of total in NG and 1.2% of total in MG, namely 3.8+/−0.9 trials in NG and 3.1+/−0.9 trials in MG). No participant exhibited behavioural or kinematics values 2.5 SDs above or below the group mean.

#### Interpersonal manipulation

We verified the reliability and efficacy of our social manipulation, as following. With regards to Visual Analogue Scales (VAS), (i) we firstly checked whether MG participants' answers to VAS2 - Reaction to manipulation confirmed our manipulation had been effective: we checked the presence of a drop-off in the expected level of cooperation quality with respect to the one rated in VAS1 - Judgments on partner personality – Pre-interaction (paired t-test VAS1–VAS2). Then, (ii) we compared data collected before and after the interaction regarding the VAS scores referred to the partner's personality and the explicit perceived similarity (i.e. two Mixed ANOVAs on Judgments on partner personality with factors Pre/Post×Neutral/Manipulated Group); the same was done on (iii) the index of implicit perceived similarity (see [69] for a detailed description of the procedure) extracted from the comparison between the self-referred BIG-5 questionnaire and the Big-5 Other-Pre and -Post (i.e. Mixed ANOVA on Implicit perceived similarity with factors Pre/Post×Neutral/Manipulated Group). After having assessed the reliability of our Interpersonal Manipulation with the analyses described above, we analysed behavioural and kinematic data from the Joint grasping Task considering “neutral” and “manipulated” couples as two separate groups. With reference to personality tests, we controlled that the two groups did not differ for baseline inter-individual differences (between-sample t-tests).

#### Joint grasping Task

Each behavioural index linked to performance at a couple-level (Accuracy, Wins and Grasping synchronicity and Start Synchronicity) was entered in a separate factorial analysis of variance (ANOVA) with Session (Session1/Session2)×Action-type (Complementary/Imitative)×Interaction-type (Free/Guided) as within-factors and Group (NG/MG) as between-factor. Concerning reaction times and maximum grip aperture (RTs, RTs Variance, MaxAp, Var_MaxAp), we run separate factorial ANOVAs with Session (Session1/Session2)×Action-type (Complementary/Imitative)×Interaction-type (Free/Guided)×Movement-type (Gross/Precise grasping) as within-subjects and Group (NG/MG) as between-subjects factor. All tests of significance were based upon an α level of 0.05. When appropriate, post-hoc tests were performed using Newman-Keuls method.

## Results

One pair of participants from the MG did not believe the Interpersonal Manipulation (as assessed by the manipulation-check procedure) and kinematic data of one pair of participants from the NG was not recorded due to technical problems. Thus, these two couples were not included in the analyses. The final sample comprised 6 pairs from the NG (12 participants) and 6 pairs from the MG (12 participants).

### Interpersonal Manipulation

The effectiveness of the social manipulation was indexed by checking several properties referred to the interaction and to the partner:

#### i) Expected cooperation

The comparison between the quality of the expected cooperation with the partner provided by MG participants (along VAS) before and after the “false-feedback exchange” (VAS1–2) showed a significant decrease in expected cooperation (paired t-test, t(11) = −3.65, *p* = .003; mPre = 71.7±8.4 mm, mPost = 46.9±18.1 mm), which indicates the participants in the MG developed a negative disposition towards their mate as consequence of the negative feedback provided by him.

#### ii) Judgments on partner personality and Explicit perceived similarity

**Figure 2:**
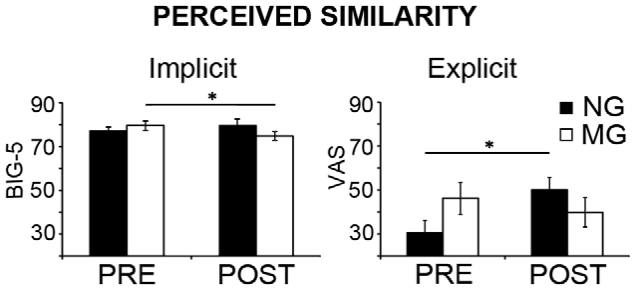
Indices of perceived similarity in the two groups before and after the interpersonal manipulation and the joint grasping task. The graphs report the indexes of Implicit (left) and Explicit (right) Perceived similarity reported by participants before (PRE) and after (POST) they underwent both the Interpersonal manipulation and the Joint grasping task. While implicit judgments extracted from the BIG-5 personality questionnaire (see main text) significantly decreased in the MG as a consequence of the Interpersonal manipulation, explicit judgements of perceived similarity (collected through a Visual Analogue Scale) significantly increased in the NG as a positive consequence of the cooperative motor interaction. Thus, both indices followed a similar pattern, though Implicit judgements were more sensitive to detect the induced negative attitude towards the partner in MG. Error bars indicate s.e.m. (*) *p*<.05.

Between samples t-tests on the ten adjectives describing the partner's personality before the interaction (and the interpersonal manipulation) confirmed that the Groups did not differ in their judgements at the beginning of the experiment (all *p*>.1_uncorr_). On the contrary, Pre-Post×Group interaction on the mean judgement about partner's personality was significant (F(1, 22) = 13.33, *p* = .001) because MG participants significantly worsened their evaluations of partner's personality (*p*<.001); this indicates they had changed their first-sight impression. Moreover, concerning the crucial question about perceived similarity (“How much do you think your partner is similar to you?”), we found a significant Pre-Post×Group interaction (F(1,22) = 7.38, *p* = .012) showing that explicit perceived similarity significantly increased (*p* = .039) only in NG (Figure 2 on the right).

#### iii) Implicit perceived similarity (BIG-5 Other -Pre and -Post)

The analysis of the implicit perceived similarity index extracted from the 25-item BIG-5 personality questionnaire complemented the explicit judgement results. Indeed, we found a significant Pre-Post×Group interaction (F(1,22) = 11.55, *p* = .002) which was accounted for by a significant reduction of implicit perceived similarity after the interaction in MG (*p* = .027) but not in NG (Figure 2 on the left).

Neither the enhancement of explicit or the reduction of implicit perceived similarity correlated (Pearson's r) with the behavioural performance or amount of won trials at the couple level (all *p*s>.3), thus ruling out the possibility that post-interaction changes in perceived similarity were influenced by the amount of won money. Importantly, t-test on the results of each personality measure (subscales in TCI, 25-item BIG-5 personality questionnaire, Eye-Test, PNR, Leadership) confirmed that group differences in Perceived Similarity ratings were not due to differences in personality traits (all *p*s>.1, See Table S1).

### Joint grasping Task

Results from the Interpersonal Manipulation procedure confirmed our social manipulation was effective and had an impact on reciprocal interpersonal perception in MG participants. Thus, we analysed behavioural and kinematic data collected during the motor task focussing on Groups' difference. Due to the high number of factors in the experimental design and the critical role of the Interpersonal Manipulation for our purposes, we extensively describe in the main text only the between factor Group significant interactions. All the other significant effects are reported in Table 1 and Table 2.

**Table 1:** All significant results on Accuracy, Grasping synchronicity and Wins.

Parameter	Effect	F	Df
**Accuracy**	*-No significant effect-*	-	-
**Grasp synchronicity**	Main effect of Session	5.45 *	1,10
	Session*Interaction-type*Group	***8.59*** *	***1,10***
**Wins**	Main effect of Interaction-type	15.88 **	1,10
**Start Synchronicity**	Main effect of Session	9.59 *	1,10
	Mani effect of Interaction-type	34.04 ***	1,10
	Main effect of Action-type	8.88 *	1,10
	Session *Action-type *Group (p = .072)	*4.05*	*1,10*
	Session*Interaction-type*Action-type*Group	***6.83*** *	***1,10***

Design: Session×Interaction-type×Action-type×Group. In bold and italics, significant effects with Group described in the main text.

(*) *p*<.05,

(**) *p*<.01,

(***) *p*<.001.

**Table 2:** All significant results on Maximum grip aperture (MaxAp) and Maximum grip aperture variance (Var_MaxAp).

Parameter	Effect	F	Df
**MaxAp**	Main effect of Interaction-type	6.9 *	1,22
	Main effect of Movement-type	650 ***	1,22
	Interaction-type*Movement-type	17.7 ***	1,22
	Action-type*Movement-type	10.3 **	1,22
	Interaction-type*Action-type*Movement-type*Group	***4.4*** *	***1,22***
	Session*Interaction-type*Movement-type*Group	***5.6*** *	***1,22***
	Session*Action-type*Movement-type*Group	***10.2*** **	***1,22***
Precise grasping only	Main effect of Interaction-type	12.0 **	1,22
	Session*Action-type*Group	***8.45*** **	***1,22***
Gross grasping only	*-No significant effect-*	-	-
**Var_MaxAp**	Main effect of Interaction-type	13.9 ***	2,22
	Main effect of Movement-type	32.42 ***	2,22
	Interaction-type*Movement-type	15.46 ***	2,22
	Session*Interaction-type*Movement-type*Group	***4.48*** *	***2,22***
Precise grasping only	Main effect of Interaction-type	15.09 ***	1,22
	Session*Interaction-type*Group	***4.7*** *	***1,22***
Gross grasping only	*-No significant effect-*	-	-

Design: Session×Interaction-type×Action-type×Movement-type×Group. Per each parameter, results from the follow-up ANOVAs are reported below the list of significant effects emerged from the general ANOVA. In bold and italics, significant effects with Group described in the main text.

(*) *p*<.05,

(**) *p*<.01,

(***) *p*<.001.

### Behavioural Data

Results related to Accuracy, Grasping Synchronicity and Wins are reported in Table 1.

Grasping Synchronicity, Wins and Accuracy (as well as Start Synchronicity, see below) are all parameters calculated at the couple-level (one value per each pair of participants) and thus the factors of the design consisted in Session×Interaction-type×Action-type×Group; indeed, the factor “Movement-type” was left outside the analysis as it was not possible to associate gross and precise grasping labels at couple-level in complementary movements, since in this condition one partner was performing a movement-type while the other was performing the opposite. As a consequence, we decided not to take the factor Movement-type into account.

#### Accuracy

No significant result emerged from the ANOVA on pairs' accuracy. Importantly, the two groups did not differ in their overall accuracy (Main effect of Group *p*>.4).

#### Grasping Synchronicity

**Figure 3:**
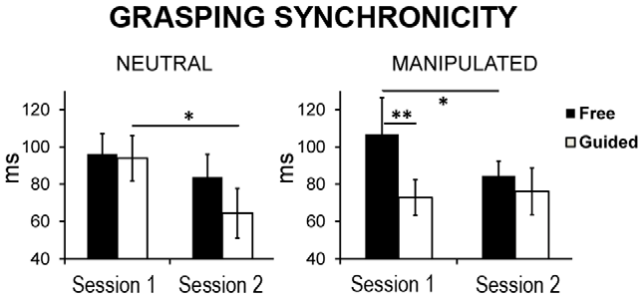
Grasping Synchronicity in the two groups in the two sessions. The graph shows that although the overall performance was comparable in the two groups, their learning profiles throughout sessions differed in the Free vs Guided interaction (significant Session×Interaction-type×Group interaction). Indeed, while NG participants improved their Grasping Synchronicity in the Guided condition, MG participants improved in the Free condition. It is worth noting that only for MG participants Free interaction was more difficult than the Guided one at the beginning of the task (Session 1). Error bars indicate s.e.m. (*) *p*<.05, (**) *p*<.01.

Although the overall performance was comparable in the two groups (Main effect of Group *p*>.9), and regardless the general improvement over sessions (Main effect of Session F(1,10) = 5.45, *p* = .042), the learning profiles of the two types of interaction (Free vs Guided) differed between the two groups as showed by the Session×Interaction-type×Group significant interaction (F(1,10) = 8.59, *p* = .015, Figure 3). Indeed, participants in the NG showed a comparable level of performance in Grasping Synchronicity between Free and Guided interactions during the first session of the motor task (as shown by the absence of any significant difference in Grasping Synchronicity in these two conditions in Session 1, *p*>.7); moreover, they improved their Grasping Synchronicity in the Guided condition throughout Session 1 and Session 2 (*p* = .02). In contrast, for MG participants the Guided interaction was easier than the Free one in Session 1 (*p* = .01); crucially, this difference vanished in Session 2 due to an improvement in Free interactions (*p* = .048).

#### Wins

Despite the differences in Grasping Synchronicity, the two Groups did not differ in terms of amount of won trials and consequently in the amount of money participants earned at the end of the experiment (Main effect of Group *p*>.4). Moreover, Wins did not show any significant interaction with the between-subjects factor Group. This was due to the wanted effect of the stair-case procedure, which let us personalize the task difficulty (i.e., the width of the tolerance time-window to assess synchronicity) to the ability in synchronising typical of each couple. As a consequence, on average, the couples of the two groups earned the same amount of money at the end of the experiment despite their performance was very dissimilar in terms of grasping synchronicity; thus, we exclude any of the reported effect could be accounted for by a systematic different level of reward.

#### Reaction Times (RTs)

The ANOVA on Reaction Times (RTs) did not show any significant interaction with the between-subjects factor Group, although the Session×Group interaction approached significance (F(1,22) = 3.67, *p* = .069). This trend was explained by the fact RTs in the NG in Session 1 tended to be longer than both NG's RTs in Session 2 (*p*<.001) and MG's ones in Session 1 (*p* = .02), and was coherent with results on RTs Variance described in Supporting Information (see Table S2 for a detailed description).

#### Start Synchronicity (Absolute difference in Reaction Times, Diff_RT)

See Table 1, lower panel, for a description of all significant results emerging from the ANOVA on Start synchronicity, i.e., on the absolute difference between partners' RTs (Diff_RT). The ANOVA showed a significant main effect of Session, Action-type and Interaction-type. Namely, trial-per-trial time-delay between participants' RTs was longer in Complementary with respect to Imitative actions (*p* = .014), was longer in Free with respect to Guided interactions (*p*<.001) and significantly decreased from Session 1 to Session 2 (*p* = .011) in both groups. However, the partners' synchronization in RTs followed different patterns in the Manipulated with respect to the Neutral group. Indeed, Diff_RT showed a trend towards significance of the Session×Action-type×Group interaction (F(1,10) = 4.05, *p* = .072). This indicates that while NG participants tended to increase their RTs synchronicity from Session 1 to Session 2 only in the Imitative condition, MG participants exhibited this tendency only in the Complementary condition. Note that the significant Session×Interaction-type×Action-type×Group quadruple interaction (F(1,10) = 6.83, *p* = .026) further specified that the reduction of Diff_RT found in the Imitative condition in NG partners was significant in both Free (*p* = .001) and Guided (*p* = .01) interaction-types. In contrast, the reduction of Diff_RT found in the Complementary condition in MG participants was significant only in Complementary-Free interactions (*p*<.001), which in this group was also the condition that in Session 1 showed the maximum Diff_RT with respect to the other conditions (all *p*s<.001).

### Kinematics data

All significant results on Maximum grip aperture (MaxAp) and Maximum grip aperture variance (Var_MaxAp) are reported in Table 2.

#### Maximum grip aperture (MaxAp)

The ANOVA on MaxAp showed that, in general, Gross grasping implied a larger grip aperture with respect to Precise grasping (*p*<.001) as it was expected given the different dimensions of the lower/upper parts of the bottle-shaped object (7 cm vs 2.5 cm of diameter). Moreover, this analysis also showed a significant main effect of Interaction-type (F(1,22) = 6.9, *p* = .016) and a significant Interaction-type×Movement-type interaction (F(1,22) = 17.7, *p*<.001; all *p*s<.001). These effects indicate that participants increased their MaxAp during Free interactions possibly to enhance the communicative value of their movements (as it has been shown by previous studies, see for instance [64]), and that this was the case for Precise grasping only, as expected given this movement implies a more careful planning and execution and on the base of previous studies showing that precise grasping is more affected by cognitive variables such as movement goals (see [63], [68] for a review).

**Figure 4:**
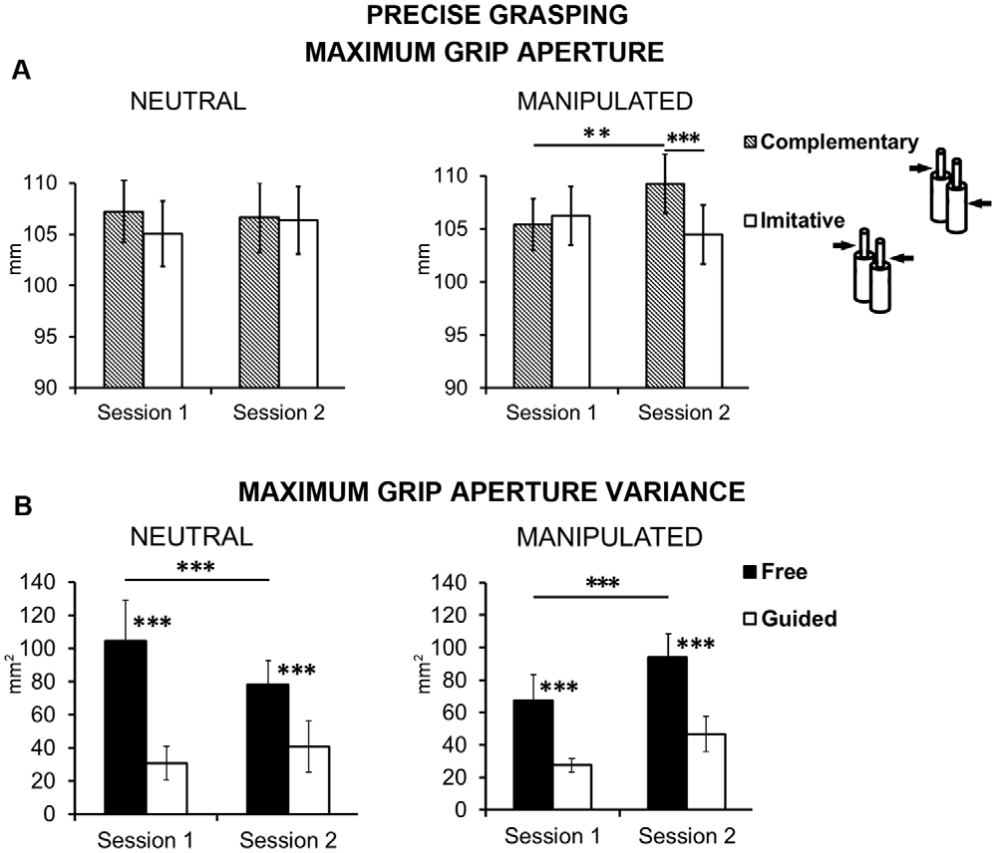
Maximum grip aperture and Maximum grip aperture variance in the two groups during Precise grasping. The upper panel (A) illustrates the four-level Session×Action-type×Movement-type×Group significant interaction shown by the general ANOVA on Maximum grip aperture (MaxAp). It indicates that, only in the MG, MaxAp of Precise grasping changed over sessions according to Action-type; indeed, only in this group, MaxAp in Complementary trials increased in Session 2 with respect to Session 1 (*p* = .006), so that the two Action-types (complementary/imitative), that were identical at the beginning of the experiment (*p* = .4), diverged in Session 2 (*p* = .001). These results suggest that in the MG interference effects, due to the observation of an incongruent movement performed by the partner, increased over time. The lower panel (B) illustrates the Session×Interaction-type×Movement-type×Group significant interaction emerged from the general ANOVA on Maximum grip aperture variance (Var_MaxAp). The grip aperture variance in Precise grasping significantly decreased in NG while it significantly increased in MG throughout sessions. These results suggest that while individuals in the NG learned how to coordinate without being influenced by the partner's movement, participants in the MG became more mutually responsive over time. This can be considered an index of the enhancement of reciprocal responsiveness between partners in the MG, in terms of both involuntary mimicry and movement corrections. The fact that these effects were found in Precise grasping only is likely to be due to the more sensitive feature of this movement-type to action-goals. Error bars indicate s.e.m. (*) *p*<.05, (**) *p*<.01, (***) *p*<.001.

Finally, this analysis showed three significant four-way interactions: Session×Interaction-type×Movement-type×Group interaction (F(1,22) = 5.6, *p* = .027), Session×Action-type×Movement-type×Group interaction (F(1,22) = 10.2, *p* = .004), and Interaction-type×Action-type×Movement-type×Group interaction (F(1,22) = 4.4, *p* = .048). Since we expected only Precise grasping to be modulated by the experimental conditions (see above) and following the main effect of Movement-type, we performed two separated ANOVAs for Gross and Precise grasps in order to make the four-way effects easier to interpret (see Table 2). As expected, the ANOVA on Gross grasping showed no significant main effect or interaction (all *p*s>.1). On the contrary, the ANOVA on Precise grasping showed again a significant main effect of Interaction-type (F(1,22) = 12.0, *p* = .002) and a significant Session×Action-type×Group interaction (F(1,22) = 8.45, *p* = .008). Post-hoc tests indicated that, only in the MG, MaxAp in Complementary actions tended to increase in Session 2 with respect to Session 1 (p = .06), so that the two Action-type (complementary/imitative), that were identical at the beginning of the experiment (*p* = .5), diverged in Session 2 (*p* = .02). This was not the case in the NG. This result also explains the two-way significant Action-type×Movement-type interaction (F(1,22) = 10.3, *p* = .004) found in the general ANOVA. Therefore it seems that Complementary actions lead participants to increase their MaxAp with respect to Imitative ones in Precise grasping (*p*<.001), and this effect seems to be a likely consequence of interference effects between self-executed and observed actions (indeed, in Complementary Precise grasping participants were performing a precise grasping while observing the partner performing a gross one). However, the higher-level interaction indicates this effect was present only in MG and only in Session 2 (Figure 4, panel A).

We suggest these results hint at the possibility that participants who underwent the interpersonal manipulation (MG), although unable to integrate the other's movements into a joint-plan, stopped being able to “ignore” the partner's movements as the interaction developed in time. As a consequence, participants started to be influenced by the partner at the expense of their individual movement execution. Notably, this visuo-motor interference was not found in NG participants.

See also Table S3 and Figure S2 for a brief description of the ANOVAs performed on normalised data (Free/Guided ratio) to further clarify the effects described above.

#### Maximum grip aperture variance (Var_MaxAp)

ANOVA on Var_MaxAp showed significant main effects of Interaction-type and Movement-type (F(1,22) = 13.9, *p*<.001 and F(1,22) = 32.42, *p*<.001, respectively) and the significant Interaction-type×Movement-type interaction (F(1,22) = 15.46, *p* = .001; all *p*s<.001) indicating that, overall, Var_MaxAp (only in Precise grasping) was higher during Free interactions when compared with Guided ones. Moreover, the significant Session×Interaction-type×Movement-type×Group interaction (F(1,22) = 4.48, *p* = .046) suggested that, during Precise grasping in Free interaction, Var_MaxAp significantly decreased from Session 1 to Session 2 in the NG (*p*<.001), while it significantly increased in the MG (*p*<.001) (see Figure 4, panel B).

As previously described for MaxAp, we divided the analysis into two separated follow-up ANOVAs for Gross and Precise grasps to further specify the 4-way significant effect (see Table 2). Again, results showed the absence of any significant effect in Gross grasping (all *p*s>.1); on the contrary, the ANOVA on Precise Grasping showed a significant main effect of Interaction-type (F(1,22) = 15.09, *p* = .001) and a significant Session×Interaction-type×Group interaction (F(1,22) = 4.7, *p* = .041). These effects confirmed that during Free interactions: i) Var_MaxAp in Precise grasping was overall higher when compared with Guided ones; and that ii) Var_MaxAp in the NG was significantly reduced from Session 1 to Session 2 (*p* = .04), while it significantly enhanced from Session 1 to Session 2 in the MG (*p* = .04).

These results suggest that while individuals in the NG learned how to improve their joint-coordination and then reduced the need of performing many individual movement corrections, MG participants increased the number of movement corrections from Session 1 to Session 2. This effect may index that mutual responsiveness increased over time for MG participants. See also Table S3 for a brief description of the ANOVAs performed on normalised data (Free/Guided ratio) to further clarify these effects.

## Discussion

In the present study we demonstrate for the first time that during on-line, face-to-face, realistic interactions, the mutual interpersonal perception heavily influences motor adjustments involved in a joint-grasping task. We assigned participants who were comparable for demographic and personality variables to one of two different experimental groups differing for the presence (manipulated group, MG) vs absence (non-manipulated neutral group, NG) of an interpersonal manipulation that negatively affected the reciprocal attitude between partners. We compared the ability of the two groups in synchronising and performing joint reach-to-grasp movements during two different interactive conditions, namely guided and free interaction. *Guided interactions* required reciprocal partners' adjustment in time only, since each individual knew what part of the object he had to grasp and was only required to adjust his movement velocity in order to be synchronous with the partner. On the contrary, *free interactions* required both time and space mutual adjustments, since participants had not only to synchronise, but also to on-line re-model their individual movements in the service of the joint-goal fulfillment (i.e., “be synchronous, but also perform imitative/complementary movements with respect to your partner's ones”).

Behavioural performance profiles showed that, while in neutral situation (NG) participants were equally challenged by the need of coordinating in free or guided interactions, participants sharing a negative interpersonal relationship (MG) were extremely skilled in guided interactions while the coordination in self-organized “free” interactive grasping requiring mutual adjustments was more demanding for them. In particular, in MG participants the difficulty in adjusting to the partner's behaviour was paralleled by a good performance in pure temporal coordination (which would benefit from neglecting the spatial features of the partner's movements in order not to be distracted by them), and by very low movement preparation and execution variability. Altogether, these data indicate that the partners in the MG tended to ignore each other and were thus impervious to mutual interference in the first session of the experiment. Crucially, the will to fulfil the joint-goal and consequently increase the individual pay-off promoted MG pearticipants' improvement in free interaction performance along the experiment (i.e., they significantly improved from session 1 to session 2). This was reflected in the second session in increased mutual interdependence and reciprocal adjustments, as indexed by higher movement variability and by the appearance of “interference effects” [19] only in MG participants.

### Simulative processes in joint-action context

Studies [16], [21], [70] indicate that performing complementary movements in joint-like situations does not imply any additional computational costs for the cognitive system with respect to performing congruent ones, and that this ability correlates with the activation of the “mirror” fronto-parietal network (see [25], [71], but also [26], [72] for same results with different accounts). Moreover, Sartori and co-authors [73]–[74] have shown that the cortico-spinal facilitation induced by action observation [75] is also found when the observed action requires a complementary response, confirming that the properties of the mirror system are not fixed but rather context- and learning-dependent ([23]–[24], [76]). Accordingly, our results showed no specific differences in performance in complementary versus imitative movements. Crucially, moreover, NG participants did not even show the typical “interference effects” between self-executed actions and those observed in the partner. It is worth noting that interference effects have been associated to “priming” effects [77] or motor simulation ([19], see also [20] for a review) underpinned by the activity of the fronto-parietal simulative “mirror” network [33]. This result expands knowledge about joint-actions, showing that, in the absence of any interpersonal manipulation, effective motor interaction is paralleled by the absence of visuo-motor interference between partners' movements. We suggest this surprising result might be sustained by the co-agents' ability to represent both their own and the partner's movements in an integrated motor plan [78], which allows each agent to predict the partner's movements so that they do not create “interference” anymore.

Moreover, we show that the improvement of MG participants in Free interactions was paralleled by an enlargement of precise grasping grip aperture in complementary (i.e. when the partner performed a gross grasping) with respect to imitative movements; these results indicate that involuntary mimicry behaviours took place in this group as the motor interaction developed in time. Notably, the presence of visuo-motor interference only in MG participants indicates the full integration of the partner's movements in the individual's motor plan was not yet fully realized.

Our results expand previous studies demonstrating that social variables influence the sensorimotor simulative processes triggered by observation of actions and painful stimulation [39]–[46], [79], and prove that the processes involved in visuo-motor simulation during a realistic interaction are affected by partners' interpersonal perception. Importantly, the temporal changes of participants' behaviour are unlikely due to a decrease of the manipulation effect since post-interaction implicit and explicit judgements showed that the negative interpersonal effect had not faded away. Rather, these results suggest that the interaction did not change the perception of the mate at an explicit “cognitive” level. Crucially, the time course of the interference effect indicates that motor interaction *per se* promotes social bonds at an implicit, sensorimotor level. Therefore, the movement of an interacting partner acts as a social “affordance” ([80], see also [67], [81]) that cannot be ignored by a co-agent once a “shared intentionality” is built [82], which in our conditions corresponded to the need of maximizing the couple pay-off.

### Entrainment and perceived similarity

Our results and experimental set-up proved adept at acquiring a bipersonal perspective. Indeed, the manipulation of the agents' *reciprocal* interpersonal perception had an impact on *both* co-agents. In view of this, we analysed the time-course of automatic entrainment as a process that considers the two partners as part of a unique dynamic system [14]. Given the sharing of the same environmental cues, we expected participants to synchronize also the behavioural parameters that were not strictly relevant to the task [13]–[14] (e.g. not only contact-times but also RTs). This is what we found in both groups as shown by the main effect of Session in the analysis of Start synchronicity. Tellingly, however, the partners' synchronization in RTs followed different patterns in the manipulated with respect to the neutral group in different experimental conditions. In particular, NG partners enhanced the synchronisation of their movement preparation timings both in free and guided interactions in the imitative condition, while MG participants did so only in the free-complementary condition. If any “entrainment” effect was to be found, it was expected to emerge in our motor task regardless the Interaction-type (i.e. both in guided and free interactions). Moreover, entrainment should be more prominent in the Imitative with respect to the Complementary conditions given that in the latter condition participants follow exactly the same trajectory and share the same environmental motor cues in terms of object affordances (i.e. their grasps are aiming at the same part of the object); thus, the selectivity of the effect found in NG is easy to interpret. On the contrary, the effect found in MG is unexpected and difficult to be explained in terms of “entrainment” processes only.

Finally, we would like to highlight that the enhancement of RTs synchronisation found between NG partners together with the evidence that only NG participants enhanced their explicit judgments about their perceived similarity with the partner is reminiscent of the influence of synchrony [49]–[50], [83] or involuntary mimicry [84]–[85] in social contexts.

### “Me & you” versus “each one on his own” motor planning strategy

We showed that in neutral realistic interactive situations (NG) two strangers are able to gradually learn how to coordinate their actions both in space and time. Moreover, when the “social bond” is disrupted by the belief that the partner has mined one's own self-esteem (MG), participants are not able to mutually coordinate in space by anticipating the partner's movements and including his actions in a smooth joint-motor plan. This is not likely to be due to attentional factors since participants were still able to achieve high-level performance when only temporal coordination was required (i.e. in Guided Interaction condition). That NG initially performed Free and Guided interactions at the same level of performance while MG did not is likely due to differences in motor planning strategies applied at the beginning of the joint-task.

In keeping with studies on imitative/complementary movements in joint-contexts [16], [21], [70], NG participants included the partner's movement in their own motor plan from the very beginning of the interaction despite the initial cost paid for monitoring the partner's movements in the Guided condition. This shows that NG participants represented the task and its goal in a highly integrated manner (what Vesper et al. [1] suggest to define a “Me+X” mode). Over time, they developed a strategy to improve performance (e.g., by reducing their RTs variability, see Table S2), and ended up entraining also their movement preparation timings. On the contrary, MG participants performed the task “everyone on his own”, as proved by the initial very high performance in Guided interaction and very low performance in the Free interaction condition, paralleled by very low RT and movement variability. However, the need to fulfil the common-goal (and thus maximize the individual pay-off) promoted the improvement of reciprocal adjustments in MG. Indeed, the improvement in Grasping synchronicity in Free interactions was paralleled by the enhancement of maximum grip aperture variance in Free interactions: this suggests the behavioural improvement was supported by an enhancement of movements corrections. Finally, the enhancement of movement corrections in Session 2 was matched with the emergence of visuo-motor interference between the self-executed actions and those observed in the partner in complementary actions. Altogether, the emergence of interference effects linked to covert imitation and the enhancement of movement variability in Free interactions indicate that co-agents enhanced social responsiveness in the second session.

Studies of face-to-face joint grasping tasks demonstrate that social factors may have an impact on action kinematics [66]–[67], [86]–[87] as well as the importance of sensorimotor simulation during coordination [88]. Moreover joint-attentional tasks [89]–[93] have investigated the role of joint-representations during interactions (see [94] for a critical review). However, to the best of our knowledge this is the first study showing that joint- (interpersonal) representations have a direct impact on the efficacy of joint-movements during tasks requiring a fully integrated representation of a joint-goal emerging from separate individual sub-goals (like for example in our Free interaction condition). Studies demonstrate that a negative interdependence between partners (e.g., a competitive context) strongly reduces the emergence of joint-representations [51]–[52]. Here we expand current knowledge by highlighting the influence of negative interdependence in a “motor” social context and its link with anticipatory motor simulation. Our paradigm allows a direct comparison between pure temporal synchronization and more complex coordination in space and time controlling for low-level movement parameters (i.e. precision and gross grasping). Thus, we provided a realistic interactive scenario, where - similarly to what happens in real-life situations -, “mutual adjustments” [78] and the prediction of both “what” the partner is doing and “when” he is going to act [5] are crucial. Moreover, our novel paradigm allows to explore the role of reciprocity between interactive agents [95]: when we properly work in concert, we adapt our behaviour to the one of another agent who is also adapting to us; this implies predictive processes that must include the possibility that my action causes a modification of the partner's action as well (“influence learning model”, [96]). In fact, when co-agents try to act “on their own”, they are not able to achieve the smooth coordination needed to fulfil effective “closed-loop” coordination [27].

## Conclusions

To sum up, we demonstrate that any joint-action implies “motor communication”. Indeed, partners' mutual adjustments are paralleled by sensitivity to partner's movements which might imply some degree of somato-motor simulation; in case a negative interpersonal perception disrupts the motor communication, sensorimotor processes are affected and a smooth integration of partners' motor plans is prevented. Thus, joint-representations are not independent from the interpersonal relation linking co-agents, proving the partner is not a “neutral” stimulus each agent needs to adapt to.

## Supporting Information

Figure S1 **The false-feedback given to participants in the manipulated group.** The VAS rating shows the feedback concerning the (false) evaluation provided by the mate that was given to each participant in manipulated pairs.
(TIF)

Figure S2 **Maximum grip aperture normalised data (Free/Guided ratio) in the two groups during Precise grasping only.** The panel A (on the left) illustrates the significant Session×Movement-type×Group interaction (F(1,22) = 7.04, *p*<.05) shown by the ANOVA on Maximum grip aperture normalised data (Free/Guided ratio). It indicates that during Precise grasping the Free/Guided ratio changed over time following opposite patterns in the two groups. More precisely, it significantly reduced in NG (*p*<.01) and it tended to increase in MG. The panel B (on the right) illustrates the significant Action-type×Movement-type×Group interaction (F(1,22) = 4.91, *p*<.05). It shows that, although the Free/Guided ratio was always higher in Precise grasping with respect to Gross grasping (Main effect of Movement-type *p*<.001), in Precise grasping it was significantly higher in complementary with respect to imitative movements only in MG (*p*<.05). The latter result suggest that -with regard to the MG- the difference in motor behaviour shown in Free vs Guided interactions may not only reflect the need of performing mutual adjustments (as it probably does in NG), but it is also due to the “noise” generated by interference effects in complementary actions. On the contrary, in the NG Free-Complementary actions were accomplished without any additional performance cost, possibly due to an alignment supported by an integrated shared representation of individuals' sub-goals. As a matter of fact, single-sample t-test showed that the only condition in which the Free/Guided ratio significantly differed from 1 was when MG performed complementary precise grasping (*p_corr_*<.05). Error bars indicate s.e.m. (*) *p*<.05, (**) *p*<.01.
(TIF)

Table S1 **Between-group t-tests on participants' personality measures.**
(DOC)

Table S2 **Supplementary results on RTs (ms) and RTs Variance (ms^2^).**
(DOC)

Table S3 **Supplementary results on normalised data (Free/Guided ratio) on Maximum grip aperture mean and variance.**
(DOC)
