# Supplementary figures and images for: And Yet They Act Together: Interpersonal Perception Modulates Visuo-Motor Interference and Mutual Adjustments during a Joint-Grasping Task
Source: PLoS One. 2012 Nov 28;7(11):e50223. doi: 10.1371/journal.pone.0050223 (PMC3509140; doi:10.1371/journal.pone.0050223)

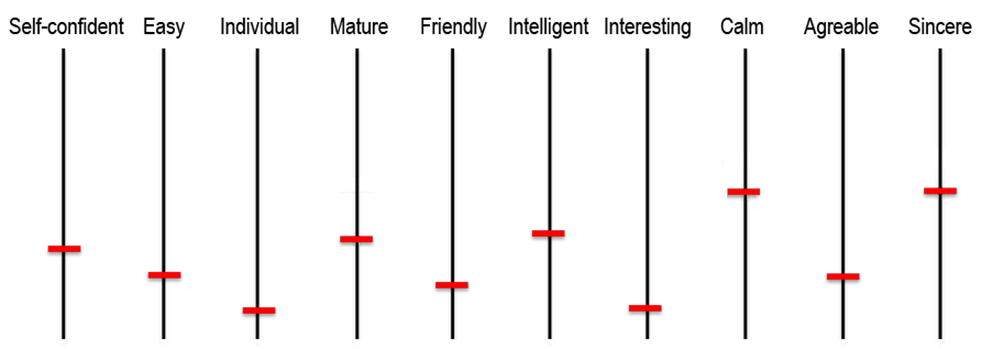

Supplement: Figure S1 — The false-feedback given to participants in the manipulated group. The VAS rating shows the feedback concerning the (false) evaluation provided by the mate that was given to each participant in manipulated pairs. (TIF) [file pone.0050223.s001.tif]

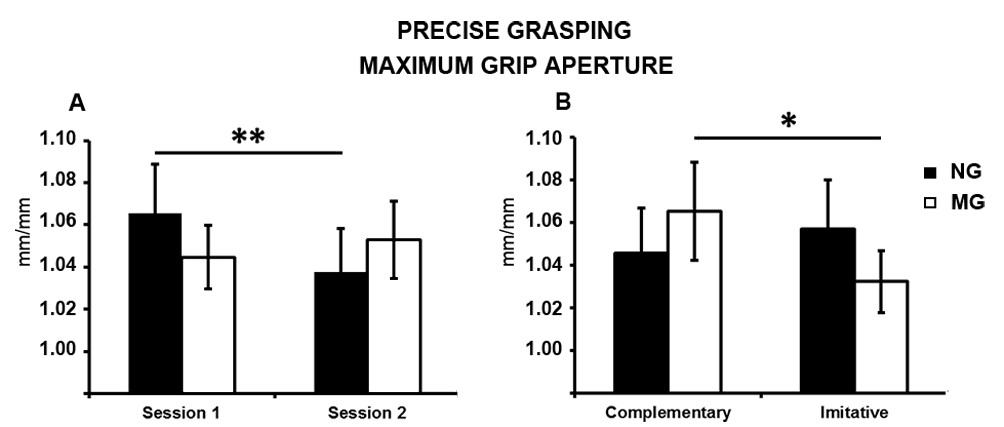

Supplement: Figure S2 — Maximum grip aperture normalised data (Free/Guided ratio) in the two groups during Precise grasping only. The panel A (on the left) illustrates the significant Session×Movement-type×Group interaction (F(1,22) = 7.04, p<.05) shown by the ANOVA on Maximum grip aperture normalised data (Free/Guided ratio). It indicates that during Precise grasping the Free/Guided ratio changed over time following opposite patterns in the two groups. More precisely, it significantly reduced in NG (p<.01) and it tended to increase in MG. The panel B (on the right) illustrates the significant Action-type×Movement-type×Group interaction (F(1,22) = 4.91, p<.05). It shows that, although the Free/Guided ratio was always higher in Precise grasping with respect to Gross grasping (Main effect of Movement-type p<.001), in Precise grasping it was significantly higher in complementary with respect to imitative movements only in MG (p<.05). The latter result suggest that -with regard to the MG- the difference in motor behaviour shown in Free vs Guided interactions may not only reflect the need of performing mutual adjustments (as it probably does in NG), but it is also due to the “noise” generated by interference effects in complementary actions. On the contrary, in the NG Free-Complementary actions were accomplished without any additional performance cost, possibly due to an alignment supported by an integrated shared representation of individuals' sub-goals. As a matter of fact, single-sample t-test showed that the only condition in which the Free/Guided ratio significantly differed from 1 was when MG performed complementary precise grasping (pcorr<.05). Error bars indicate s.e.m. (*) p<.05, (**) p<.01. (TIF) [file pone.0050223.s002.tif]
