# Supplementary material for: And Yet They Act Together: Interpersonal Perception Modulates Visuo-Motor Interference and Mutual Adjustments during a Joint-Grasping Task
Source: PLoS One. 2012 Nov 28;7(11):e50223. doi: 10.1371/journal.pone.0050223 (PMC3509140; doi:10.1371/journal.pone.0050223)
Supplement: Table S1 — Between-group t-tests on participants' personality measures. (DOC) [file pone.0050223.s003.doc]

**Table S1. Between-group t-tests on participants’ personality measures.**

|  | ***TCI*** | ***Big-5*** | ***Eye-Test*** | ***Leadership*** | ***PNR*** |
| --- | --- | --- | --- | --- | --- |
| ***T-test (p)*** | All *p*s > .16 | All *p*s > .4 | *p* = .8 | *p* = .3 | All *p*s > .5 |

The comparison of participants’ personality profiles showed no significant differences between the Neutral and the Manipulated group.
