# Supplementary material for: And Yet They Act Together: Interpersonal Perception Modulates Visuo-Motor Interference and Mutual Adjustments during a Joint-Grasping Task
Source: PLoS One. 2012 Nov 28;7(11):e50223. doi: 10.1371/journal.pone.0050223 (PMC3509140; doi:10.1371/journal.pone.0050223)
Supplement: Table S2 — Supplementary results on RTs (ms) and RTs Variance (ms2). (DOC) [file pone.0050223.s004.doc]

**Table S2. Supplementary results on RTs (ms) and RTs Variance (ms^2^).**

| **Parameter** | **Effect** | **F** | **Df** |
| --- | --- | --- | --- |
| **RTs** | Main effect of Session | 37.08*** | 1,22 |
|  | Main effect of Interaction-type | 7.5* | 1,22 |
|  | Main effect of Action-type | 4,66* | 1,22 |
|  | Session*Interaction-type | 12.5** | 1,22 |
|  | *Session * Group (p = .069)* | *3.67* | *1,22* |
| **RTs variance** | Main effect of Session | 6.69* | 1,22 |
|  | Main effect of Interaction-type | 15.31*** | 1,22 |
|  | Session*Interaction-type | 4.49* | 1,22 |
|  | Interaction-type*Action-type*Movement-type*Group | 5.22* | 1,22 |
|  | ***Session*Group*** | ***6.11**** | ***1,22*** |

The ANOVA on RTs Variance showed a significant Session x Group interaction (F(1,22) = 6.11; *p* = .022) which was accounted for by the fact that RTs Variance was higher in NG with respect to MG in Session 1 (RTs Variance in Session 1, NG vs MG, *p* = .009) and then significantly reduced from Session 1 to Session 2 (RTs Variance in NG in Session 1 vs Session 2, *p* =.008).

As a reduction of variance of a behavioural parameter is considered to be an index of its increased predictability (Vesper et al. (2011) “Making oneself predictable: reduced temporal variability facilitates joint action coordination”. Exp Brain Res 211: 517–530), these results suggest that NG increased the predictability of their RTs from Session 1 to Session 2 while, on the contrary, the MG did not show such a trend. We suggest the between group difference in RT variability time-patterns (i.e. from Session 1 to Session 2) might be due to the fact that only NG participants were sensitive to the partner’s movements in Session 1 and tried to coordinate their RTs with a consequent enhancement of RT variability. On the contrary, MG participants disregarded the partner’s movements (together with the partner’s RTs) and adhered to their own idiosyncratic movement preparation timing, and this kept low the RTs variability in MG in the first session. Coherently, RT variance was higher in Free vs Guided interaction only in NG, supporting the hypothesis that the increase in RT variance reflected the attempt to coordinate (Interaction-type*Action-type*Movement-type*Group). Crucially, it has to be noted that the higher RTs variability found in NG in Session 1 was not due to an unspecific between-group difference in RT variability since the two groups did not differ on mean RT variance (main effect of Group, p > .1). Finally, these results on RT Variance were coherent with the trend emerged from the analysis of mean RTs, which showed that the Session x Group interaction approached significance (*p* = .069). Indeed, this trend indicates that RTs in NG in Session 1 were not only more variable but also significantly longer than both NG’s RTs in Session 2 (*p* < .001) and MG’s ones in Session 1 (*p* = .02): this supports the hypothesis NG participants were initially trying to coordinating their movement preparation with the partner’s one and then chose to become more predictable as a coordination strategy.
