# Supplementary material for: And Yet They Act Together: Interpersonal Perception Modulates Visuo-Motor Interference and Mutual Adjustments during a Joint-Grasping Task
Source: PLoS One. 2012 Nov 28;7(11):e50223. doi: 10.1371/journal.pone.0050223 (PMC3509140; doi:10.1371/journal.pone.0050223)
Supplement: Table S3 — Supplementary results on normalised data (Free/Guided ratio) on Maximum grip aperture mean and variance. (DOC) [file pone.0050223.s005.doc]

**Table S3. Supplementary results on normalised data (Free/Guided ratio) on Maximum grip aperture mean and variance.**

| **Parameter** | **Effect** | **F** | **Df** |
| --- | --- | --- | --- |
| **MaxAp** | Main effect of Movement-type | 18.26*** | 1,22 |
|  | ***Action-type*Movement-type*Group*** | ***4.91**** | ***1,22*** |
|  | ***Session*Movement-type*Group*** | ***7.04**** | ***1,22*** |
| **Var_MaxAp** | Main effect of Movement-type | 18.12*** | 1,22 |
|  | ***Session*Group*** | ***4.59**** | ***1,22*** |
|  | ***Session*Action-type*Group*** | ***5.93**** | ***1,22*** |
|  | ***Session*Action-type* Movement-type*Group*** | ***4.66**** | ***1,22*** |

We performed a separate analysis normalising (dividing) the kinematic data from the Free interaction condition on the data from the Guided one in order to clarify the impact of the need to perform “mutual adjustments” controlling for movement and time-coordination constrains. In other words, we wanted to have a clear index showing the impact of the manipulation on the ability of individuals to perform adjustments according to the partner’s behaviour. Thus, the factors in the mixed ANOVA didn’t include anymore the factor Interaction-type (Free/Guided) and the analysis had Session (Session1/2) x Action-type (Complementary/Imitative) x Movement-type (Gross/Precise grasping) as within-couple factors and Group (NG/MG) as between-couples factor. The table reports all significant effects. In bold and italics, significant interactions with the between-subjects factor Group. (*) *p* < .05, (**) *p* < .01, (***) *p* < .001.

See Figure S2 for a detailed description of the significant interactions on Maximum grip aperture. With regards to movement variance, this analysis showed a Session x Group, Session x Action-type x Group and a Session x Action-type x Movement-type x Group significant interaction. Namely, only in Precision grasping, the Free/Guided ratio of Imitative movement significantly reduced in NG (*p* < .001) while it tended to increase in the MG. Moreover, while the Free/Guided ratio was different from 1 in the NG since the beginning (*p_corr_* < .05 both in Session 1 and 2), in the MG it started to differ from 1 only in Session 2 (*p_cor_*_r_ < .01). These results indicate that the need to perform mutual adjustments had an impact on the kinematics of the MG only in Session2; namely, the need to improve Free interaction performance led participants in this group to try to perform movement corrections: as a consequence, in Session 2 movement variance enhanced with respect to Guided interactions.
